# Reporting of Ethical Considerations in Qualitative Research Utilizing Social Media Data on Public Health Care: Scoping Review

**DOI:** 10.2196/51496

**Published:** 2024-05-17

**Authors:** Yujie Zhang, Jiaqi Fu, Jie Lai, Shisi Deng, Zihan Guo, Chuhan Zhong, Jianyao Tang, Wenqiong Cao, Yanni Wu

**Affiliations:** 1 Nanfang Hospital Southern Medical University Guangzhou China; 2 School of Nursing Southern Medical University Guangzhou China

**Keywords:** qualitative research, informed consent, ethics approval, privacy, internet community

## Abstract

**Background:**

The internet community has become a significant source for researchers to conduct qualitative studies analyzing users’ views, attitudes, and experiences about public health. However, few studies have assessed the ethical issues in qualitative research using social media data.

**Objective:**

This study aims to review the reportage of ethical considerations in qualitative research utilizing social media data on public health care.

**Methods:**

We performed a scoping review of studies mining text from internet communities and published in peer-reviewed journals from 2010 to May 31, 2023. These studies, limited to the English language, were retrieved to evaluate the rates of reporting ethical approval, informed consent, and privacy issues. We searched 5 databases, that is, PubMed, Web of Science, CINAHL, Cochrane, and Embase. Gray literature was supplemented from Google Scholar and OpenGrey websites. Studies using qualitative methods mining text from the internet community focusing on health care topics were deemed eligible. Data extraction was performed using a standardized data extraction spreadsheet. Findings were reported using PRISMA-ScR (Preferred Reporting Items for Systematic Reviews and Meta-Analyses Extension for Scoping Reviews) guidelines.

**Results:**

After 4674 titles, abstracts, and full texts were screened, 108 studies on mining text from the internet community were included. Nearly half of the studies were published in the United States, with more studies from 2019 to 2022. Only 59.3% (64/108) of the studies sought ethical approval, 45.3% (49/108) mentioned informed consent, and only 12.9% (14/108) of the studies explicitly obtained informed consent. Approximately 86% (12/14) of the studies that reported informed consent obtained digital informed consent from participants/administrators, while 14% (2/14) did not describe the method used to obtain informed consent. Notably, 70.3% (76/108) of the studies contained users’ written content or posts: 68% (52/76) contained verbatim quotes, while 32% (24/76) paraphrased the quotes to prevent traceability. However, 16% (4/24) of the studies that paraphrased the quotes did not report the paraphrasing methods. Moreover, 18.5% (20/108) of the studies used aggregated data analysis to protect users’ privacy. Furthermore, the rates of reporting ethical approval were different between different countries (*P*=.02) and between papers that contained users’ written content (both direct and paraphrased quotes) and papers that did not contain users’ written content (*P*<.001).

**Conclusions:**

Our scoping review demonstrates that the reporting of ethical considerations is widely neglected in qualitative research studies using social media data; such studies should be more cautious in citing user quotes to maintain user privacy. Further, our review reveals the need for detailed information on the precautions of obtaining informed consent and paraphrasing to reduce the potential bias. A national consensus of ethical considerations such as ethical approval, informed consent, and privacy issues is needed for qualitative research of health care using social media data of internet communities.

## Introduction

Social media are web-based computer-mediated tools to collaborate, share, or exchange information, ideas, pictures, or videos in virtual communities and networks such as message boards, communities, chat rooms, forums, Twitter, and Facebook [1]. Moreover, patients and researchers can use internet communities to provide health care and disseminate health information [2,3]. Health care refers to the efforts made to improve or maintain physical, mental, or emotional well-being, including prevention, diagnosis, treatment, recovery, and other physical and mental impairments [4]. Currently, with 57% of the global population’s access to social media, more than 40% of the patients and caregivers worldwide utilize the internet community for health care information needs [5]. With diverse populations accessing internet communities and sharing information about health care topics, researchers have the opportunity to collect and analyze text about health care from a diverse range of participants in the internet community, which was unavailable previously [6]. Usually, quantitative data are derived from information extraction, which can be analyzed statistically, and the summary results presented cannot be directly linked to individual participants. In contrast, qualitative research within internet community analysis posts and comments qualitatively or thematically involves a more detailed and in-depth analysis and understanding of the full written content [7]. However, a controversial ethical problem has been raised about conducting qualitative research containing internet users’ verbatim quotes that could lead to traceability of the original post, thereby causing a threat to an individual’s privacy [8]. Additionally, a previous study investigated public and patients’ views regarding ethics in research using social media data and reported that internet users were aggrieved if they found any of their quotes cited in a medical research paper without obtaining their informed consent [9]. Further, besides the privacy breach caused by posts being traced, there is greater harm for special groups or vulnerable groups if we do not highlight the importance of the technical standards for text mining and privacy protection in health care. For instance, some unusual postings, abnormal pictures, and interactions that were expressed by individuals with mental disorders in social media can be detected by researchers by using text mining tools without obtaining their consent [10]. The publication of research on mental disorders, including quotes in posts, can result in a high risk of information harm, which can lead to personal information being revealed and further stigmatization of the condition or disease [11]. Since 2001, ethical concerns have been debated for decades about ethical approval, informed consent, and how to ensure anonymity and preserve data privacy and confidentiality in qualitative research in the internet community [12-14].

With the rapid development of social media and internet research, some ethical guidelines or standards have been published to ensure that research based on internet communities is conducted ethically. The Association of Internet Researchers (internet research ethical guidelines 2.0 and 3.0) showed that researchers working without the direct approval of ethics review boards would have additional challenges to face, and obtaining informed consent is obviously impracticable in several big data projects. However, with the ethical issues about privacy breaches and harms of risk of discrimination, the Association of Internet Researchers recommended reserving the acquisition of informed consent to the dissemination stage by asking for informed consent from specific participants before publication of their quotes [15,16]. Furthermore, researchers should take responsibility for information confidentiality and anonymity according to the internet research ethics criteria prepared by the National Committee for Research Ethics in the Social Sciences and the Humanities guidelines, which recommend a basic research ethic norm for the analyses, reports, and evaluations that apply to all research [17]. Moreover, the National Committee for Research Ethics in the Social Sciences and the Humanities guidelines contain more details about the demand for legal consent and privacy standards imposed by the European Union’s General Data Protection Regulation. The General Data Protection Regulation is a European Union–wide regulation targeting the project of personal data processing. The General Data Protection Regulation defines personal data as any information relating to an identifiable person (data subject), including name, online identification number, location data, and other factors related to personal, physical, physiological, mental, or social identity [18]. The General Data Protection Regulation recommends using anonymous data and deleting identifiable information to ensure the confidentiality of the data. Consent should be obtained from the individual for use in scientific research [18,19]. The British Psychological Society guideline does not explicitly refer to the internet community but suggests that researchers may consider paraphrasing the verbatim quotes to reduce the risk of being traced or identified in qualitative research [20]. When paraphrasing, steps must be put into place to ensure that the original meaning of the message is maintained. Currently, there is no widespread consensus on ethical considerations by social media researchers.

Some researchers have tried to explore the reporting of existing ethical considerations in research papers using social media data. For instance, Sinnenberg et al [6] reported that only 32% and 12% of the papers mentioned acquiring ethical approval and informed consent, respectively, by utilizing multiple analysis methods, including surveillance, intervention, recruitment, engagement, content analysis, and network analysis with Twitter data before 2015. Thereafter, Takats et al [21] conducted an updated examination based on Sinnenberg et al’s [6] study. They found that of 367 studies using different methodological approaches, including sentiment mining, surveillance, and thematic exploration of public health research using Twitter data between 2010 to 2019, 17% of the studies included verbatim tweets and identifiable information about the internet users [21]. Similarly, Lathan et al [22] reviewed papers, including both qualitative and quantitative methods, by using Facebook data to explore public health issues and reported that only 48% and 10% of the papers obtained ethical approval and informed consent, respectively. Furthermore, in a study on research using YouTube data or comments, Tanner et al [23] found that only 26.1% of these studies sought ethical approval, only 1 paper (0.08%) sought informed consent, and 27.7% contained identifiable information. These findings indicate widespread neglect of ethical issues such as ethical approval, informed consent, and privacy issues in research papers using social media data.

Our study focuses on the ethical challenges of qualitative studies utilizing social media data. First, social media can be considered as sources for qualitative data collection because of the low cost, vast amount of available sources about health information, and users’ health behaviors, experiences, and attitudes. Second, qualitative research is context-dependent and mainly contains quotations and written content to support the viewpoint. It is acknowledged that quote materials from social media would potentially be traced back to the original posts and threaten the users’ privacy [24]. This is supported by findings reported by Ayers et al [25] who found that online searches of verbatim Twitter quotes in journal papers described as “content analyses” or “coded Twitter postings” can be traced back to individual internet users 84% of the time. Furthermore, Lathan et al [22] identified that 46% of the studies with verbatim or paraphrased quotes could be traced to the original posts in 10 minutes. Therefore, it is essential to investigate the extent to which ethical oversight is reported in qualitative studies using social media data. Moreover, qualitative research often involves personally sensitive data about health conditions and diseases; hence, anonymity and proper deidentification would be more important for researchers [26,27].

Previous studies have reviewed the ethical challenges and methodological use of social media platforms such as Twitter [6,21], Facebook [22], and YouTube [23] for health care research in both qualitative and quantitative studies. Although there is plenty of qualitative data pouring into social media such as blogs, Twitter, Facebook, and Weibo, evidence is lacking on the investigation of ethical considerations targeting qualitative data in different software and web-based discussion forums to provide a more comprehensive understanding of the ethical issues. To address the ethical considerations in qualitative research of different internet communities and draw the attention of researchers and publishers to ethical issues, we conducted this study to evaluate the ethical practices and ethical considerations of qualitative studies on health care by using data of internet communities. This review aims to (1) assess the rates of reporting institutional review board (IRB) approval and informed consent in studies focused on mining text in the internet community and social media, (2) compare these rates according to the year of publication, country conducting the research, website included in the study’s analysis, and journal’s guidelines about ethical approval for the type of study, and (3) describe whether the studies used anonymized/deidentified data.

## Methods

### Research Design

We conducted a scoping review to investigate how qualitative research mining social media data handles ethical approval, informed consent, and confidential issues. We performed this study according to the PRISMA-ScR (Preferred Reporting Items for Systematic Reviews and Meta-Analyses Extension for Scoping Reviews) guidelines. The completed PRISMA-ScR checklist is provided in Multimedia Appendix 1.

### Search Strategy

All published qualitative studies from 2010 to March 31, 2023, focusing on mining text from online community and social media sources about health care in the following databases were included in this study: PubMed, Web of Science, CINAHL, Cochrane, and Embase. A standardized search string containing Medical Subject Headings (MeSH) and non-MeSH entry terms was used in the search strategy. In addition, the reference lists of the retrieved papers and citation tracking were manually searched as a supplement to database searches to improve comprehensiveness. Gray literature was also identified through internet searches in Google Scholar and OpenGrey websites. The search strategies are represented in Multimedia Appendix 2.

### Inclusion and Exclusion Criteria

We divided the criteria into 2 parts. First, we limited the inclusion and exclusion criteria used at the title and abstract screening stage eligible for (1) studies mining existing text and posts from the internet community and social media data focusing on health care topics, (2) studies using qualitative methods or available qualitative parts in mixed methods studies to analyze data, and (3) studies only written in English. Ineligible studies were those related to investigating the use and dissemination of social media in health care, using social media or internet community as an intervention tool, and using social media to conduct web-based interviews, surveys, or focus groups. We also excluded studies published as reviews, case studies, conference abstracts, commentaries, policies, guidelines, and recommendations. Second, at the full screening stage, the specific eligible inclusion criteria were studies focused on mining text about health care topics with full-text papers. Studies that did not have the full text after contacting the authors and that were not originally in the English language were excluded.

### Study Selection

All results of the searches were entered into the EndNote library, and duplicates were removed. Two researchers reviewed the titles and abstracts based on the inclusion and exclusion criteria independently. Those studies that were irrelevant to the study topic were discarded, and then the full text was screened to select eligible papers. Any disagreements were discussed and resolved by consensus or a third person.

### Data Extraction

Data were extracted between April 2023 and May 2023. Two researchers independently read the full text carefully, and the results were extracted using a standardized data extraction spreadsheet, including research type, first author, study objective, sample size, publication time, country where the research was conducted or country of the first author, website or internet community the studies focus on, type of data collected from social media, language of collected posts or data, privacy level of data (public or privacy posts), study design, research results, published journal, and information about the ethical considerations. Disagreements were resolved by consensus of a third person. The information about ethical considerations was analyzed to investigate the rates of reporting ethical approval, informed consent, and privacy issues: whether IRB review was reported (IRB approval, IRB exemption, unnecessary, not mentioned) and the reason for not requiring IRB approval; whether informed consent was obtained from participants or the websites’ administrators, consent types (digitally informed consent or written informed consent, informed consent is not required, consent was waived by IRB), and the methods used to obtain consent in each study; and whether quoting a post in papers could lead to the identification of internet users in each study. The description of users’ posts (verbatim quote, paraphrase) was recorded. We also analyzed if posts were paraphrased to maintain the original meaning, if actions were taken to deidentify the internet users, and if the posts contained other identifying information (ie, usernames, photos, links, hashtags) attached to the post. As every journal would provide publication ethical considerations and requirements, we also searched the submission guidelines and editorial policies of each journal submission website to check whether the journal contained any ethical guidance targeting studies using data from internet community and social media platforms. Additional information was included about the details of ethical approval, informed consent, and privacy, for example, whether individuals can withdraw their quotes if they want to be excluded from the study at any time without any reprisal and whether the quotations were tested for deidentification via search engines. There was excellent agreement on the primary outcome between the 2 researchers (k>.95 for all).

### Data Analysis

Data were analyzed using SPSS software (IBM Corp). The chi-square test or Fisher exact tests (when cell size was less than 5) were used to test for differences between the rates of informed consent and ethical approval according to publication year, website, and different countries. All *P* values were 2-sided, and *P* values <.05 indicated significance.

## Results

### Study Selection for the Review

**Figure 1:**
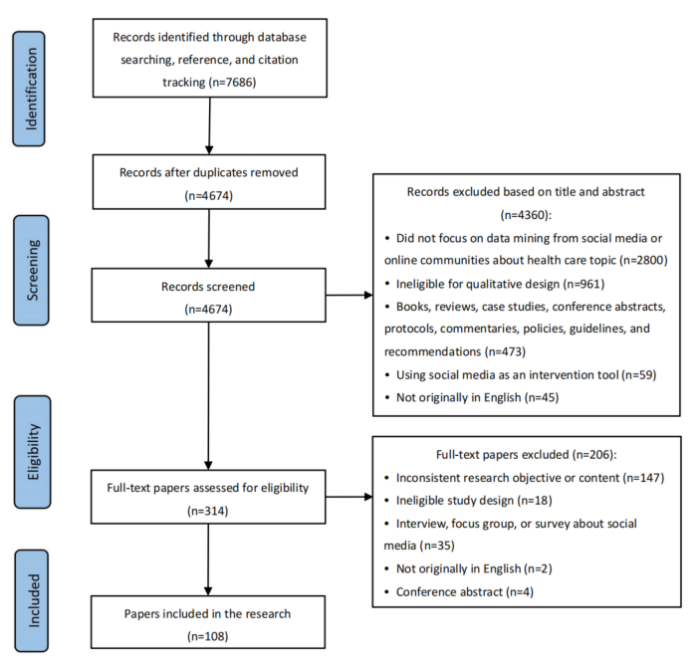
PRISMA-ScR (Preferred Reporting Items for Systematic Reviews and Meta-Analyses Extension for Scoping Reviews) flow diagram of the study selection process.

We reviewed 4674 papers after removing the duplicates. After screening the titles, abstracts, and full-texts, we reviewed 108 eligible papers (Figure 1). The full list of the included papers and all the extracted information are incorporated in Multimedia Appendix 3 [28-135]. Of the 108 studies reviewed, 73 (67.6%) were qualitative studies and 35 (32.4%) were mixed methods studies. All papers had text mined from internet communities or social media for qualitative analysis. The sample size ranged from 32 to 392,962. Approximately 82.4% (89/108) of the studies were published after 2018, and there was a sharp increase in the number of studies from 2019 to 2022. Moreover, nearly half of the studies (55/108, 50.9%) were published in the United States. Regarding the websites for mining text, the most widely used social media platform was Twitter (42/108, 38.9%), followed by Facebook (17/108, 15.7%).

### Ethics Approval in These Studies

Our results indicated that of the 108 studies, 78 (72.2%) reported ethics approval. Of the 78 studies, 31 (40%) explicitly stated that ethics approval was obtained before the study was undertaken, 33 (42%) reported that the ethics approval was granted through exemptions by the local IRB, and 14 (18%) explicitly demonstrated that approval by the ethics committee was not required because publicly available data were collected from internet communities and social media platforms. However, 30 (27.8%) of the 108 studies did not mention about obtaining IRB approval (Table 1).

**Table 1:** Ethical considerations in the qualitative studies using data of the internet community.

Ethical considerations		Values, n (%)
**Institutional review board review sought (N=108)**		
	Yes (including exemption)	64 (59.3)
	No	14 (12.9)
	Not mentioned	30 (27.8)
**Informed consent (N=108)**		
	Yes	14 (12.9)
	No (not required/exemption)	35 (32.4)
	Not mentioned	59 (54.7)
**Anonymous data (N=108)**		
	Yes	104 (96.3)
	No	4 (3.7)
**Studies contain internet users’ written content (n=76)**		
	Verbatim quote	52 (68)
	Paraphrased	24 (32)
**Identifiable information attached to the post (links, photos, screenshots) (n=76)**		
	Yes	14 (18)
	No	62 (82)

Based on our exploration of the ethical guidelines of each journal to determine whether there were ethical requirements for studies mining social media data, only 36.1% (39/108) of the studies were published in journals that required ethical considerations for studies gathering data from social media platforms by using internet and digital technologies. Of the 39 studies published in 19 journals, 27 (69%) were published in the *Journal of Medical Internet Research* and its sister journals. The submission guidelines of the *Journal of Medical Internet Research* state that authors of manuscripts describing studies of internet, digital tools, and technologies are required to verify that they have adhered to local, national, regional, and international laws and regulations, and are required to verify that they complied with informed consent guidelines. Moreover, 2 journals also provided a specific requirement, that is, when researchers interact with individuals or obtain privacy information gathered from social media platforms, they should obtain ethics approval prior to conducting the study and informed consent from anyone who could potentially be identified. Surprisingly, there were no significant differences in the ethics approval reportage between journals with ethics approval guidelines and those that did not have ethics guidelines for researchers gathering data from social media platforms (*P*=.08). Notably, the rates of reporting ethics approval were different between different countries (*P*=.02). However, there were no statistically significant differences between the rates of reporting ethical approval and different websites or publication years (all *P*>.05) (Table 2).

**Table 2:** Reporting of ethical considerations in studies published in different publication years, countries, websites, and journals containing ethical requirements for research involving text mining and internet users’ written content.

Items (total number of studies)			Ethical approval reported									Informed consent reported						
			Values, n (%)		Chi-square *(df)*			*P* value			Values, n (%)			Chi-square *(df)*			*P* value	
**Year**						17.2 (13)			.11						12.1 (13)			.52
	2010 (n=1)	1 (100)								0 (0)								
	2011 (n=2)	2 (100)								1 (100)								
	2012 (n=2)	1 (50)								1 (50)								
	2013 (n=2)	0 (0)								0 (0)								
	2014 (n=3)	1 (33)								2 (67)								
	2015 (n=2)	1 (50)								1 (50)								
	2016 (n=3)	3 (100)								2 (67)								
	2017 (n=4)	4 (100)								3 (75)								
	2018 (n=12)	7 (58)								3 (25)								
	2019 (n=9)	5 (56)								4 (44)								
	2020 (n=24)	16 (67)								9 (38)								
	2021 (n=14)	11 (78)								5 (36)								
	2022 (n=25)	22 (88)								16 (64)								
	2023 (n=5)	4 (80)								2 (40)								
**Country conducting the research**						28.4 (20)			.02						17.8 (20)			.64
	United States (n=55)	40 (73)								23 (43)								
	Australia (n=12)	10 (83)								6 (50)								
	United Kingdom (n=8)	8 (100)								5 (62)								
	Canada (n=9)	7 (78)								5 (56)								
	China (n=3)	0 (0)								0 (0)								
	Netherlands (n=3)	2 (67)								2 (67)								
	Turkey (n=2)	2 (100)								1 (50)								
	United Arab Emirates (n=2)	1 (50)								1 (50)								
	India (n=2)	0 (0)								0 (0)								
	Sweden (n=1)	1 (100)								1 (100)								
	Norway (n=1)	1 (100)								1 (100)								
	Italy (n=1)	1 (100)								1 (100)								
	Germany (n=1)	1 (100)								0 (0)								
	France (n=1)	1 (100)								0 (0)								
	Finland (n=1)	1 (100)								1 (100)								
	Bangladesh (n=1)	1 (100)								1 (100)								
	Austria (n=1)	1 (100)								1 (100)								
	Thailand (n=1)	0 (0)								0 (0)								
	Saudi Arabia (n=1)	0 (0)								0 (0)								
	Singapore (n=1)	0 (0)								0 (0)								
	Israel (n=1)	0 (0)								0 (0)								
**Website cited in the research**						14.7 (11)			.12						18.7 (11)			.07
	Twitter (n=42)	26 (62)								14 (33)								
	Facebook (n=17)	12 (70)								10 (59)								
	≥2 websites (n=14)	11 (79)								6 (43)								
	Reddit (n=9)	8 (89)								3 (33)								
	Specialist forums (n=7)	7 (100)								5 (57)								
	Instagram (n=5)	4 (80)								4 (80)								
	Blog (n=4)	4 (100)								4 (100)								
	YouTube (n=4)	3 (75)								1 (25)								
	Sina Weibo (n=3)	0 (0)								0 (0)								
	Quora (n=1)	1 (100)								1 (100)								
	STUMPPI (n=1)	1 (100)								1 (100)								
	WhatsApp (n=1)	1 (100)								1 (100)								
**Whether journals contained ethical requirements for research involving text mining from internet community and social media platforms**						3.5 (1)			.08						2.2 (1)			.16
	Yes (n=39)	24 (62)								14 (36)								
	No (n=69)	54 (78)								35 (51)								
**Whether studies had users’ written content**						12.9 (1)			<.001						2.2 (1)			.15
	Yes (n=76)	60 (79)								38 (50)								
	No (n=32)	14 (44)								21 (67)								

### Informed Consent

Of the 108 studies, 59 (54.7%) showed that they did not include any information about informed consent and 49 (45.3%) mentioned informed consent. Of the 49 studies that mentioned informed consent, 14 (13%) demonstrated that informed consent was waived by local institutional boards, and 21 (19%) reported that informed consent was not required because this information is publicly available in websites or did not involve human participants. We interpreted this as not seeking informed consent. Only 14 (12.9%) of the 108 studies explicitly indicated that informed consent was obtained (Table 1). Among the 14 studies, 2 (14%) only provided a generic statement that informed consent was obtained but did not report the process of how the informed consent was obtained, while 12 (86%) received digital informed consent. Of the 12 studies that reported receiving digital informed consent, 6 reported that they sought permission from the communities’ or groups’ administrators and by posting a statement of the research objective on the group’s wall, while 5 studies contacted the participants privately via email, commenting below the posts and software to gain consent, and 1 study reported that it had sent a digital version of the informed consent book. Furthermore, among the studies that had obtained informed consent, 7 studies included the statement that the individuals’ posts would be removed if they wanted to be excluded from the study, and they could withdraw from the study whenever they wanted. In addition, the rates of reporting informed consent showed no statistical significance between publication years, different countries, and different websites (all *P*>.05) (Table 2).

### Confidentiality of the Information

All data sources were obtained from anonymous websites or communities, and the majority (104/108, 96.3%) of the data sources did not contain usernames. Notably, only 3.7% (4/108) of the studies contained the participants’ usernames or pseudonyms. One study reported that pseudonyms like Sasha had been used instead of the real name. The other 3 studies contained the expression for usernames but did not state whether pseudonyms were used. Except for 9 studies that used nonnative language quotes and 3 studies that were transcribed into text via video, among the 108 included studies, 76 (70.3%) quoted at least one native language post in their reports. Additionally, 20 studies presenting aggregated analysis or composite accounts did not include any quotation or written content. Of the 76 studies containing internet users’ written content, 52 (68%) contained just verbatim-quoted participants’ posts and 24 (32%) contained paraphrased posts (Table 1). Among the 52 studies containing direct and verbatim quotations, which are likely to be traced to the original posts from users, only 17 (33%) studies took measures to deidentify the users. The 17 studies mentioned that all names or usernames were removed and personal identifying information was removed to maintain privacy, while 42% (22/52) of the studies did not mention any measures that were taken to deidentify the users and maintain confidentiality. Approximately 32% (24/76) of the studies described that they paraphrased posts and removed any explicitly identified personal information to maintain confidentiality to reduce the likelihood of users being identified via search engines. Of the 24 studies, 20 (83%) reported that the quotations were slightly modified or summarized for readability, the symbol information was removed using “…”, and key identifiable information was removed to protect privacy while maintaining the meaning of posts. Four of the 24 (17%) studies did not report the methods and details of paraphrasing. Notably, only 3% (2/76) of the studies containing users’ written content showed that researchers intentionally entered each quote into search engines to ensure that every quote did not lead to the original posts. Moreover, of the 76 studies containing written content, 62 (82%) did not contain other types of identity information attached to the posts, while 14 (18%) included other identifying data (hashtags, emojis, geolocation, photos, links, screenshots) attached to the original posts for analysis (Table 1). Of the 14 studies including other identifying information, 4 (29%) contained photos and screenshots associated with the website pages. Of the 52 studies that disclosed verbatim quotes and other identifiable information, 26 (46%) studies reported informed consent consideration, and only 8 (15%) obtained explicitly informed consent. Additionally, of the 77% (40/52) of the studies that mentioned IRB or ethical review, 38% (15/40) received IRB approval, and 63% (25/40) of the studies were granted exemption. The proportion of reporting ethical approval in studies containing users’ written content was modestly higher than that in studies not containing users’ written content (60/76, 79% vs 14/32, 44%; *P*<.001) (Table 2).

## Discussion

### Principal Findings and Comparison to Prior Work

In this scoping review, we included 108 studies (Multimedia Appendix 3; [28-135]) that focused on mining text from internet community and social media data for health care research, and we reviewed the ethical consideration reportage and outcome reports in these studies. We found that the rates of reporting IRB approval and informed consent in qualitative research on health care utilizing social media data were 59.3% (64/108) and 12.9% (14/108), respectively. Our findings demonstrate that the key ethical considerations for qualitative research in online communities are insufficiently discussed and described. However, the reporting rates of ethical considerations in the papers in our scoping review were much higher than those reported in systematic reviews including multiple analysis methodologies on only 1 social media platform. For example, ethics approval and informed consent were reported in 48% and 10% of research studies using only Facebook data [22], 32% and 0% from 2006 to 2019 [21], 40% and 0.9% (only 1 paper) from 2015 to 2016 in public health research using only Twitter data [25], and 26.1% and 0.8% (only 1 paper) in researches incorporating only YouTube data [23], respectively. In fact, previous studies were limited to only a few selected websites such as Twitter, Facebook, and YouTube. There is a lack of research that incorporates a variety of different social media data for comparisons. Differences in the reporting of ethical considerations may be attributed to the different methodologies adopted by studies. For example, Lathan et al [22] analyzed the ethical considerations in studies including predictive or model development, while our research focuses on the ethical considerations in qualitative studies.

Importantly, our findings indicate that there is a need to develop a standardized and apparent approach for the reporting of ethical considerations in qualitative research of data from social media and online communities. Our research demonstrates that the rates of reporting ethics approval are different in different countries (*P*=.02). Specifically, a wide variety of national research ethics governing bodies and over 1000 laws, regulations, and standards provide oversight for human subjects research in 130 countries. Obviously, a guideline is needed for best ethical practices for qualitative research involving posts from social media platforms. Surprisingly, there were no significant differences between the rates of reporting ethical approval and those of journals specifying ethical requirements for studies involving text mining (*P*=.08). This inconsistent result of publication guidelines and reports of ethical approval consent is similar to previous findings on the ethical standards in COVID-19 human studies [136]. Although there are journal publication guidelines for studies mining social media data, the reports of ethical approval and consent in the papers published in such journals do not exactly follow the guidelines. Consequently, this finding indicates that more ethical awareness is needed among researchers, editors, and reviewers for qualitative studies on data mining.

Besides the different legal and regulations in different countries, the inconsistency in the ethics approval in published papers may be because social media research is a highly interdisciplinary science, and computer science researchers may be less experienced or may pay less attention to the key ethical issues of protecting human subjects [137]. Medical and health science researchers may have considered some ethical concerns about gathering social media data but they may not be familiar with the relevant guidelines. For example, the Association of Internet Researchers has a detailed ethical guideline targeting social scientists conducting digital research, while it may be less popular and less well-known among medical and health care researchers. At the institute level, Ferretti et al [138] noticed that institutionalized review committees, especially the individual IRB institutes for universities and health care systems lack knowledge about the methodology, text mining technical standards, data security, and ethical harms for studies using big data and social media as sources. Because of this lack of knowledge, institutional ethics committees may have inconsistent ethical criteria and perspectives about web-based projects using social media data [139]. Therefore, some ethics review committees exclude research on internet communities from ethical oversights because their ethics standards are confined only to medical fields. Above all, it is additionally challenging for ethical approval institutions because of the continuous development and dynamic change of studies using social media data. Furthermore, it is necessary for ethics committee members to be trained about the ethical issues in studies mining text from social media. Inviting interdisciplinary researchers to join in the approval process would be an appropriate method to increase the awareness of ethical considerations [140,141].

Interestingly, the reporting rate of obtaining informed consent for mining social media data in qualitative studies was unexpected. The most influential ethical reports such as the Nuremberg Code [142], Declaration of Helsinki [143], and the Belmont Report [144] have demonstrated the principle of informed consent in research involving humans. Our review shows that only 12.9% (14/108) of the studies explicitly obtained informed consent and 32.4% (35/108) of the studies reported that informed consent was exempted by IRB or was not required, as the information was available publicly in websites or did not involve human participants. Our results are similar to those of Wongkoblap et al [145] who reported that only 16.7% of the studies received informed consent from participants prior to data analysis on data mining of social network data on mental health disorders.

There are multiple reasons for the challenges in obtaining informed consent in an internet setting. First, it is impractical for researchers to gain individual informed consent from a large number of users in an internet community [146]. Second, members of ethics review boards lack consensus about the need for informed consent from an internet community for qualitative research under the current legal definition [147]. Moreover, there has been a debate on the criteria of human subject research in using social media data. The federal regulation recommends that if data in the studies are obtained from public social media websites, where data are identifiable and do not require interaction with individuals, such studies do not constitute human subject research, while studies involving the identification of private information or interaction with the individual can be considered as human subject research [148]. In contrast, some researchers believe that social media and big data research are not ethically exceptional and should be treated in the same manner and with the same rules as those for traditional forms of research [149]. There is ambiguity as to what is appropriate or should be standard practice for obtaining informed consent.

Currently, it is challenging to maintain privacy and protect the traceability of individuals posting content in the internet community. Our findings indicated that 70.3% (76/108) of the studies contained internet users’ written content, of which 68% (52/76) included verbatim quotations of users’ posts that could lead to identification, and 18% (14/76) of the studies included other identifiable information such as links, screenshots, and emojis linked to original posts, which are similar to the findings of Ayers et al [25] and Lathan et al [22]. Usha Lawrance et al [150] and Wilkinson and Thelwall [151] argued that using direct quotes to support findings would lead to the identification of users and breach users’ confidentiality in internet community data. Moreover, quoting social media posts or disclosing usernames violate the International Committee of Medical Journal Editors’ ethics standards, which state that identifying information such as written descriptions and photos should not be published unless the information is essential for scientific purposes and the participants give written informed consent for publication [152]. Furthermore, our study demonstrates that the proportion of studies containing users’ written content (both direct and paraphrased quotations) is higher than that of studies that do not include any quotation or written content (60/76, 79% vs 14/32, 44%; *P*<.001)——a tentative explanation is that some researchers realized that ethical reportage should be stricter for qualitative papers with quotations from social media posts due to privacy and security issues. This is supported by Boyd and Crawford [153] who stated that rigorous thinking about the process of mining and anonymizing big data is required for ethics boards to ensure that people are protected. Our findings show that 32% (24/76) of the studies intentionally paraphrased the quotes to ensure that users could not locate them, and 20 studies used aggregated data interconnected with anonymity. Moreover, it is recommended by Wilkinson and Thelwall [151], Bond et al [154], and Markham et al [155] that researchers should not directly quote and work with aggregate data sets and separate texts from their original context, which is more acceptable to participants. In addition, the British Psychological Society guidelines recommend that researchers consider paraphrasing any verbatim quotes to reduce the risk of these being traced to the source [20]. Notably, 13 of the 25 papers in this study showed that they did not report the precautions taken for paraphrasing. This may be due to the lack of detailed methodology and consensus about paraphrasing quotes to reduce bias and maintaining the original meaning.

### Limitations and Strengths

Our scoping review has several limitations. First, our research was limited to qualitative studies and the qualitative parts in mixed methods studies on text mining from social media, and it is unclear whether ethical considerations are critical in quantitative studies among internet communities. Second, we were restricted to studies published in English language and those with the full text available, and therefore, we could be underestimating the number of relevant papers published in other languages. Third, the rates of reporting ethical approval, informed consent, and privacy of this research relied on self-reported data. Thus, it is possible that although certain studies did not report the process of ethical considerations, such considerations may have been followed during the research. Conversely, some studies may have mentioned about the ethical considerations but may not have conducted them in practice. Hence, there is a bias because of the lack of accurate documentation that must be considered.

### Conclusion

Social media text mining can be a useful tool for researchers to understand patient experiences of health conditions and health care. However, as illustrated by the absence of ethical discourse in publications, our analysis indicates significant gaps in the ethical considerations and governance of qualitative research of internet posts. Therefore, a complete and consistent consensus guideline of ethical considerations in qualitative research of internet posts is needed to protect users’ data. With the continued advancing development of text-mining techniques, qualitative studies mining text from social media should be more cautious while using user quotations to maintain user privacy and protect the traceability of the internet users posting content. We suggest that authors should report their results by using aggregated findings or deidentified ways like paraphrasing instead of verbatim quotations, which can prevent internet users from being identified through search engines. In addition, authors should provide more detailed information about the precautions taken for obtaining informed consent and paraphrasing to reduce the potential bias. Furthermore, journals and editors should pay more attention to the reporting standards of ethical consideration and privacy issues in qualitative research involving social media data.

## Appendix

Multimedia Appendix 1 PRISMA-ScR (Preferred Reporting Items for Systematic Reviews and Meta-Analyses Extension for Scoping Reviews) checklist.

Multimedia Appendix 2 Search strategy for each database.

Multimedia Appendix 3 Summary of included literature.

## Abbreviations

IRB: institutional review board
MeSH: Medical Subject Headings
PRISMA-ScR: Preferred Reporting Items for Systematic Reviews and Meta-Analyses Extension for Scoping Reviews

## Data Availability

All data extracted and analyzed during this study are presented in this paper and in the multimedia appendices.
