# Supplementary material for: Reporting of Ethical Considerations in Qualitative Research Utilizing Social Media Data on Public Health Care: Scoping Review
Source: J Med Internet Res. 2024 May 17;26:e51496. doi: 10.2196/51496 (PMC11143395; doi:10.2196/51496)
Supplement: Multimedia Appendix 2 [file jmir_v26i1e51496_app2.docx]

**Multimedia Appendix 2. Search strategy for each database**

1. **Search strategy for *PubMed***

#1 social media [Mesh Terms] 15191

#2 “social network site*” [Title/Abstract] 340

#3 “social media” [Title/Abstract] 27365

#4 Facebook [Title/Abstract] 6290

#5 twitter [Title/Abstract] 6294

#6 tweet [Title/Abstract] 901

#7 linkedin [Title/Abstract] 329

#8 instagram [Title/Abstract] 1716

#9 weibo [Title/Abstract] 436

#10 whatsapp [Title/Abstract] 1547

#11 telegram [Title/Abstract] 184

#12 wechat [Title/Abstract] 1038

#13 YouTube [Title/Abstract] 2862

#14 Reddit [Title/Abstract] 549

#15 Tumblr [Title/Abstract] 68

#16 hashtag* [Title/Abstract] 940

#17 “public comment” [Title/Abstract] 388

#18 blog [Title/Abstract] 1041

#19 “internet communit*” [Title/Abstract] 91

#20 “online communit*” [Title/Abstract] 1405

#21 #1 OR #2 OR #3 OR #4 OR #5 OR #6 OR #7 OR #8 OR #9 OR #10 OR #11 OR #12 OR #13 OR #14 OR #15 OR #16 OR #17 OR #18 OR #19 OR #20 41193

#22 [Delivery of Health Care](https://www.ncbi.nlm.nih.gov/mesh/68003695) [Mesh Terms] 1219645

#23 “[Delivery of Health Care](https://www.ncbi.nlm.nih.gov/mesh/68003695)” [Title/Abstract] 12032

#24 “health care” [Title/Abstract] 450381

#25 nurs* [Title/Abstract] 526421

#26 “health management” [Title/Abstract] 7861

#27 #21 OR #22 OR #23 OR #24 OR #25 1867935

#28 Empirical research [MeSH Terms] 85418

#29 “Empirical research” [Title/Abstract] 7757

#30 “qualitative research” [Title/Abstract] 33069

#31 “qualitative study” [Title/Abstract] 58922

#32 interview [Title/Abstract] 166176

#33 “content analysis” [Title/Abstract] 41442

#34 “Thematic analysis” [Title/Abstract] 36596

#35 phenomenology [Title/Abstract] 11391

#36 “focus group” [Title/Abstract] 33830

#37 “grounded theory” [Title/Abstract] 14714

#38 ethnography [Title/Abstract] 4461

#39 “Case study” [Title/Abstract] 96468

#40 “discourse analysis” [Title/Abstract] 2483

#41 #26 OR #27 OR #28 OR #29 OR #30 OR #31 OR #32 OR #33 OR #34 OR #35 OR #36 OR #37 OR #38 447859

#42 #21 AND #27 AND #41 1938

***2.Sea*rch strategy for *Web of Science***

#1 TS=social media 126110

#2 TI=social network site* [253](https://www.webofscience.com/wos/alldb/summary/fa7613e8-4272-43ff-93f7-e8c5b8f842d3-7a8e7bbd/relevance/1)

#4 TI=online community 2431

#5 TI=internet community 463

#5 TI=facebook [3,309](https://www.webofscience.com/wos/alldb/summary/2ffc303b-4774-4408-82da-4e861440ee01-7a8e7ff9/relevance/1)

#6 TI=twitter 4856

#7 TI=tweet 1582

#8 TI=linkedin [65](https://www.webofscience.com/wos/alldb/summary/c8749b3a-6894-43b0-bf84-1de9f5da654e-7a8e83eb/relevance/1)

#9 TI=instagram [1,091](https://www.webofscience.com/wos/alldb/summary/cfc2c715-a83e-4510-9a69-7026d12fe483-7a8e85fb/relevance/1)

#10 TI=weibo [280](https://www.webofscience.com/wos/alldb/summary/06a9d8be-4573-4366-9853-25623d780e32-7a8e8822/relevance/1)

#11 TI=whatsapp [500](https://www.webofscience.com/wos/alldb/summary/9786b189-ac42-425f-8811-92732a379151-7a8e8c55/relevance/1)

#12 TI=telegram [78](https://www.webofscience.com/wos/alldb/summary/2067b241-f525-43af-a9cb-e3a1100f2d65-7a8e8e5b/relevance/1)

#13 TI=wechat 509

#14 TI=Youtube 2714

#15 TI=Reddit 249

#16 TI=Tumblr 20

#17 TI=hashtag 201

#18 TI=public comment 874

#19 TI=blog 939

#20 #1 OR #2 OR #3 OR #4 OR #5 OR #6 OR #7 OR #8 OR #9 OR #10 OR #11 OR #12 OR #13 OR #14 OR #15 OR #16 OR #17 OR #18 OR #19 136511

#21 TS=[Delivery of Health Care](https://www.ncbi.nlm.nih.gov/mesh/68003695) [2354](https://www.webofscience.com/wos/alldb/summary/ad78a512-e487-4ffb-b3a0-fe686c8eb975-7a8ee500/relevance/1)03

#22 TS=health care 1146973

#23 TS=nurs* [9486](https://www.webofscience.com/wos/alldb/summary/ff781506-92fd-4d90-a828-0510b1d02ea4-7a8ee81a/relevance/1)92

#24 TS=health management [147](https://www.webofscience.com/wos/alldb/summary/46660558-6ea0-4076-b40c-7e4e959c1228-7a8ee993/relevance/1)01

#25 #21 OR #22 OR #23 OR #24 1985314

#26 TS=empirical research [1247](https://www.webofscience.com/wos/alldb/summary/9da7ddb4-fbf0-4210-a030-2e1a499f5365-7a8f1843/relevance/1)00

#27 TS=qualitative research 219431

#28 TS=qualitative study 400864

#29 TS=interview 622879

#30 TS=content analysis 65490

#31 TS=Thematic analysis 41913

#32 TS=phenomenology 35902

#33 TS=focus group 45853

#34 TS=grounded theory 20993

#35 TS=ethnography 10846

#36 TS=Case study [4476](https://www.webofscience.com/wos/alldb/summary/28553057-d73a-4d30-9202-2cdce328c383-7a8f3146/relevance/1)70

#37 TS=discourse analysis 7824

#38 #26 OR #27 OR #28 OR #29 OR #30 OR #31 OR #32 OR #33 OR #34 OR #35 OR #36 OR #37 1513394

#39 #20 AND #25 AND #38 3909

1. **Search strategy for *CINAHL***

#1 SU social media 20923

#2 TI social network site 150

#3 TI Internet community 107

#4 TI Online community 814

#5 TI facebook 2644

#6 TI twitter 1688

#7 TI tweet 1223

#8 TI linkedin 89

#9 TI instagram 524

#10 TI weibo 38

#11 TI whatsapp 161

#12 TI telegram 14

#13 TI wechat 170

#14 TI Youtube 840

#15 TI Reddit 108

#16 TI Tumblr 15

#17 TI hashtag 98

#18 TI public comment 144

#19 TI blog 1321

#20 #1 OR #2 OR #3 OR #4 OR #5 OR #6 OR #7 OR #8 OR #9 OR #10 OR #11 OR #12 OR #13 OR #14 OR #15 OR #16 OR #17 OR #18 OR #19 17937

#21 AB delivery of health care 15668

#22 AB health care 369874

#23 AB nurs* 388914

#24 AB health management 20645

#25 #21 OR #22 OR #23 OR #24 689,384

#26 AB Empirical research 5360

#27 AB qualitative research 21528

#28 AB qualitative study 61361

#29 AB interview 216795

#30 AB content analysis 34472

#31 AB Thematic analysis 31690

#32 AB phenomenology 4424

#33 AB focus group 45753

#34 AB grounded theory 13979

#35 AB ethnography 2678

#36 AB Case study 114784

#37 AB discourse analysis 2780

#38 #26 OR #27 OR #28 OR #29 OR #30 OR #31 OR #32 OR #33 OR #34 OR #35 OR #36 OR #37 296,063

#39 #20 AND #25 AND #38 450

1. **Search strategy for Embase**

#1 social media/exp 44115

#2 ‘social network site*’:ti,ab,kw 411

#3 ‘social media’:ti,ab,kw 43991

#4 facebook:ti,ab,kw 6285

#5 twitter:ti,ab,kw 6290

#6 tweet:ti,ab,kw 1117

#7 linkedin:ti,ab,kw 329

#8 instagram:ti,ab,kw 1714

#9 weibo:ti,ab,kw 436

#10 whatsapp:ti,ab,kw 1544

#11 telegram:ti,ab,kw 183

#12 wechat:ti,ab,kw 1038

#13 Youtube:ti,ab,kw 4081

#14 Reddit:ti,ab,kw 640

#15 Tumblr:ti,ab,kw 83

#16 hashtag*:ti,ab,kw 1316

#17 ‘public comment’:ti,ab,kw 486

#18 blog:ti,ab,kw 1601

#19 ‘internet communit*’:ti,ab,kw 115

#20 ‘online communit*’:ti,ab,kw 1814

#21 #1 OR #2 OR #3 OR #4 OR #5 OR #6 OR #7 OR #8 OR #9 OR #10 OR #11 OR #12 OR #13 OR #14 OR #15 OR #16 OR #17 OR #18 OR #19 OR #20 61223

#21 ‘[delivery of health care](https://www.ncbi.nlm.nih.gov/mesh/68003695)’:ti,ab,kw 4580

#22 ‘health care’:ti,ab,kw 546756

#23 nurs*:ti,ab,kw 632188

#24 ‘health management’:ti,ab,kw 9694

#25 #12 OR #13 OR #14 OR #15 1119146

#26 ‘Empirical research’/exp 7194

#27 ‘qualitative research’/exp 111939

#28 ‘Empirical research’:ti,ab,kw 8688

#29 ‘qualitative study’:ti,ab,kw 68066

#30 interview:ti,ab,kw 218727

#31 ‘content analysis’:ti,ab,kw 47147

#32 ‘Thematic analysis’:ti,ab,kw 44393

#33 phenomenology:ti,ab,kw 14192

#34 ‘focus group’:ti,ab,kw 41628

#35 ‘grounded theory’:ti,ab,kw 18291

#36 ethnography:ti,ab,kw 5125

#37 ‘Case study’:ti,ab,kw 117707

#38 ‘discourse analysis’:ti,ab,kw 2865

#39 #26 OR #27 OR #28 OR #29 OR #30 OR #31 OR #32 OR #33 OR #34 OR #35 OR #36 OR #37 OR #38 547341

#40 #21 AND #25 AND #39 1075

1. **Search strategy for Cochrane Library**

#1 MeSH descriptor: [social media] explode all trees 435

#2 “social network site*”:ti,ab,kw 3

#3 “social media”:ti,ab,kw 2582

#4 facebook :ti,ab,kw 909

#5 twitter:ti,ab,kw 219

#6 tweet:ti,ab,kw 32

#7 linkedin:ti,ab,kw 16

#8 instagram:ti,ab,kw 191

#9 weibo:ti,ab,kw 11

#10 whatsapp:ti,ab,kw 643

#11 telegram:ti,ab,kw 106

#12 wechat:ti,ab,kw 477

#13 Youtube:ti,ab,kw 211

#14 Reddit:ti,ab,kw 19

#15 Tumblr:ti,ab,kw 1

#16 hashtag*:ti,ab,kw 33

#17 “public comment”:ti,ab,kw 144

#18 blog:ti,ab,kw 104

#19 “internet community”:ti,ab,kw 10

#20 “online community”:ti,ab,kw 109

#21 #1 OR #2 OR #3 OR #4 OR #5 OR #6 OR #7 OR #8 OR #9 OR #10 OR #11 OR #12 OR #13 OR #14 OR #15 OR #16 OR #17 OR #18 OR #19 OR #20 4032

#22 MeSH descriptor: [[Delivery of Health Care](https://www.ncbi.nlm.nih.gov/mesh/68003695)] explode all trees 57532

#23 “health care”:ti,ab,kw 73209

#24 nurs*:ti,ab,kw 52445

#25 “health management”:ti,ab,kw 565

#26 #22 OR #23 OR #24 OR #25 154508

#27 MeSH descriptor: [Empirical Research] explode all trees 1985

#28 “Empirical research”:ti,ab,kw 1806

#29 “qualitative research”:ti,ab,kw 9293

#30 “qualitative study”:ti,ab,kw 17292

#31 interview:ti,ab,kw 27097

#32 “content analysis”:ti,ab,kw 1679

#33 “Thematic analysis”:ti,ab,kw 2544

#34 phenomenology:ti,ab,kw 203

#35 “focus group”:ti,ab,kw 2546

#36 “grounded theory”:ti,ab,kw 421

#37 ethnography:ti,ab,kw 60

#38 “Case study”:ti,ab,kw 3208

#39 “discourse analysis”:ti,ab,kw 45

#40 #27 OR #28 OR #29 OR #30 OR #31 OR #32 OR #33 OR #34 OR #35 OR #36 OR #37 OR #38 OR #39 46999

#41 #21 AND #26 AND #40 182

**Search strategy used for searching grey literature**

1. **Search strategy for Opengrey**

(“social media” OR Facebook OR twitter OR tweet OR linkedin OR instagram OR weibo OR whatsapp OR telegram OR wechat OR YouTube OR Reddit OR Tumblr OR hashtag OR “public comment” OR blog OR “internet community” OR “online community”) AND (“Delivery of Health Care” OR “health care” OR nurs* OR “health management”) AND (“Empirical research” OR “qualitative research” OR “qualitative study” OR interview OR “content analysis” OR “Thematic analysis” OR phenomenology OR “focus group” OR “grounded theory” OR ethnography OR “Case study” OR “discourse analysis”) 125

1. **Search strategy for Google Scholar**

(social media OR “social media” OR Facebook OR twitter OR tweet OR linkedin OR instagram OR weibo OR whatsapp OR telegram OR wechat OR YouTube OR Reddit OR Tumblr OR hashtag OR “public comment” OR blog OR “internet community” OR “online community”) AND (Delivery of Health Care OR "Delivery of Health Care" OR "health care" OR nurs* OR "health management") AND (Empirical research OR “Empirical research" OR “qualitative research” OR “qualitative study” OR interview OR “content analysis” OR “Thematic analysis” OR phenomenology OR “focus group" OR “grounded theory” OR ethnography OR “Case study” OR “discourse analysis”) 7
